# 4345 Two-step Algorithm for Clostridioides difficile is Inadequate for Differentiating Infection from Colonization in Children

**DOI:** 10.1017/cts.2020.442

**Published:** 2020-07-29

**Authors:** Maribeth R Nicholson, Jacob M Parnell, Irtiqa Fazili, Sarah C. Bloch, D. Borden Lacy, Eric Skaar, Kathryn M Edwards

**Affiliations:** 1 Vanderbilt University Medical Center

## Abstract

OBJECTIVES/GOALS: In 2017, new guidelines recommended multi-step algorithms for CDI diagnosis, and clinical centers rapidly implemented changes despite limited pediatric data. We assessed a multi-step algorithm using NAAT followed by EIA for ability to differentiate symptomatic CDI from colonization in children. METHODS/STUDY POPULATION: We prospectively enrolled pediatric patients with cancer, cystic fibrosis, or inflammatory bowel disease who were not being tested or treated for CDI and obtained a stool sample for NAAT. If positive by NAAT (colonized), EIA was performed. Children with symptomatic CDI who tested positive by NAAT via the clinical laboratory were also enrolled and EIA performed on residual stool. A functional cell cytotoxicity neutralization assay (CCNA) was performed in addition. RESULTS/ANTICIPATED RESULTS: Of the 138 asymptomatic children enrolled, 24 (17%) were colonized. An additional 37 children with symptomatic CDI were enrolled. Neither EIA positivity (41% versus 21%, *P* = 0.11) or CCNA positivity (49% versus 46%, *P* = 0.84) were significantly different between symptomatic versus colonized children. When both EIA and CCNA were positive, children were more commonly symptomatic than colonized (33% versus 13%, *P* = 0.04). DISCUSSION/SIGNIFICANCE OF IMPACT: A multi-step testing algorithm with NAAT and EIA failed to differentiate symptomatic CDI from colonization in our pediatric cohort. As multi-step algorithms are moved into clinical care, pediatric providers will need to be aware of the continued limitations in diagnostic testing.

